# Endodontic Management of a Two-rooted Mandibular First Premolar with Five Root Canals with Cone-beam Computed Tomography: A Case Report

**DOI:** 10.30476/DENTJODS.2020.83376.1049

**Published:** 2021-09

**Authors:** Ahmad Nouroloyouni, Mehrdad Lotfi, Amin Salem Milani, Sarah Nouroloyouni

**Affiliations:** 1 Dept. of Endodontics, Faculty of Dentistry, Ardabil University of Medical Sciences, Ardabil, Iran; 2 Dept. of Endodontics, Faculty of Dentistry, Tabriz University of Medical Sciences, Tabriz, Iran; 3 Dept. of Pediatric Dentistry, Faculty of Dentistry, Ardabil University of Medical Sciences, Ardabil, Iran

**Keywords:** Anatomic variations, Cone-beam computed tomography, Endodontic treatment, Mandibular first premolar, Tooth morphology

## Abstract

Proper knowledge of the anatomic structure of the root canal system is a vital prerequisite for successful root canal therapy. This report presents the endodontic management a two-rooted lower first premolar with five root canals. A similar case has not been reported to date. The use of cone beam computed tomography (CBCT) in rare and doubtful cases helps establish an accurate diagnosis and render successful endodontic treatment thereafter. This article helps broaden our knowledge about the possible anatomic diversities as to teeth with more roots and root canals than expected normally.

## Introduction

The success of root canal treatment hinges on a thorough knowledge of the root and root canal system morphology and the imaging techniques used to identify all the canals, especially in the lower premolar teeth [ 1 - 2 ].

The majority of mandibular first premolars have one root and one root canal. However, some rather rare variations like two-rooted, three-rooted, and four-rooted premolars have been reported in the literature [ 3 ]. 

Slowey reported that the mandibular premolars are the most complicated teeth concerning endodontic treatment [ 4 ]. A study reported the highest failure rate in the mandibular first premolars [ 5 ]. A large number of endodontic failures of mandibular premolars have been cited as evidence [ 5 - 6 ]. 

A possible reason for these failures is the diversity of anatomic variations, which dental practitioners often have limited knowledge about. Hereby, after obtaining informed consent from the patient we report, for the first time, the root canal treatment of a two-rooted mandibular first premolar with five root canals to help broaden the range of possible anatomic variations in the mandibular premolars.

## Case Presentation

**Figure 1:**
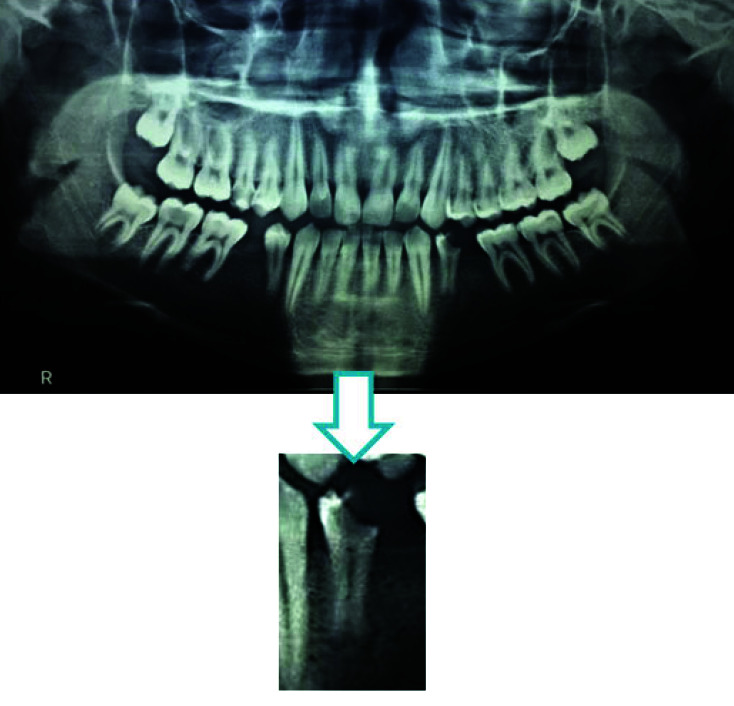
Panoramic view

**Figure 2:**
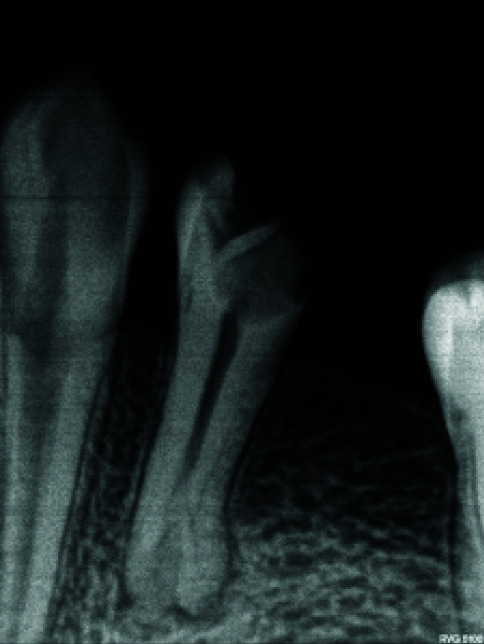
Preoperative diagnostic radiograph

An 18-year-old male with no systemic disease referred to the Department of Endodontics for endodontic treatment of the mandibular left first premolar. After obtaining informed consent from the patient, the diagnostic tests were carried out. The tooth exhibited prolonged sensitivity to cold test and tenderness to percussion; the electric pulp test yielded a positive response. Preoperative radiographic examination revealed deep caries, widening of the periodontal ligament, and an unusual root canal system anatomy (Figures 1 and 2).

**Figure 3:**
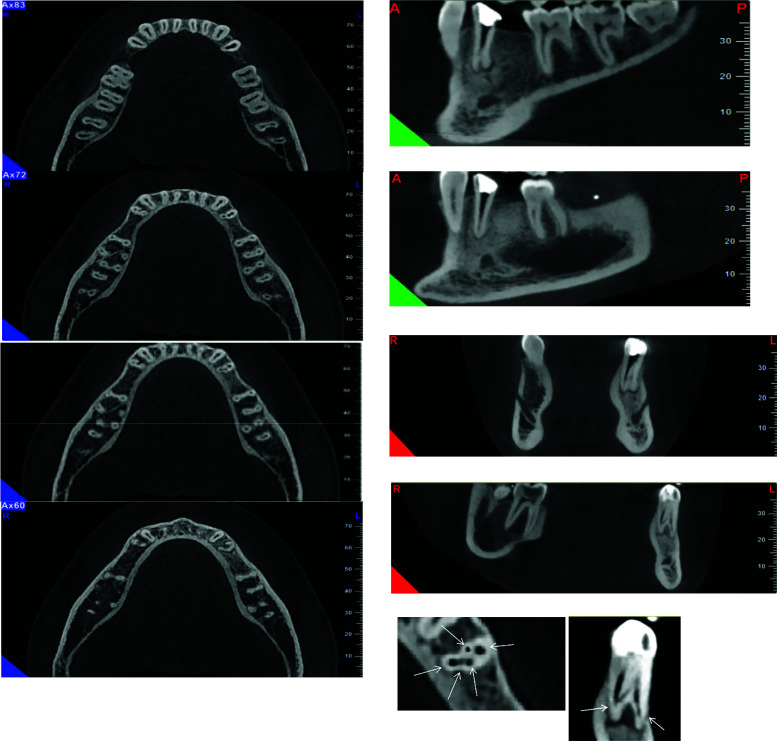
CBCT (Cone-beam Computed Tomography) images showing the roots from different planes

**Figure 4:**
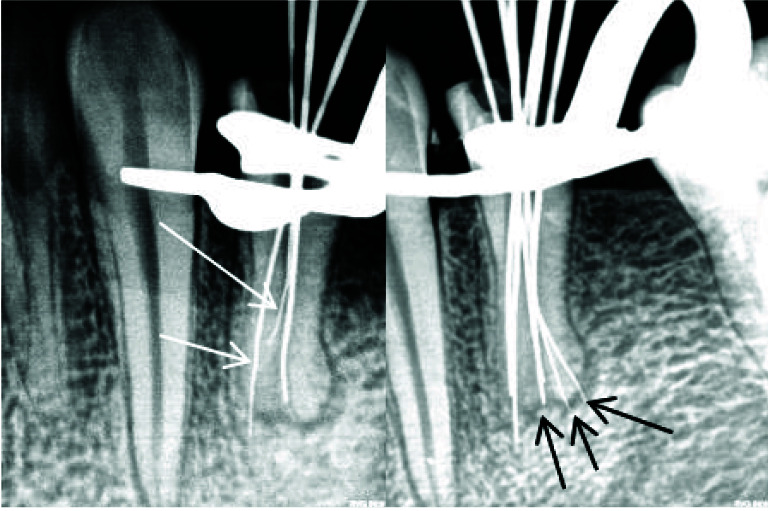
Working length determination radiograph

Cone beam computed tomography (CBCT) examinations were administered after informed consent for further evaluation of this rare and complicated root canal anatomy three-dimensionally. CBCT examination of the lower jaw was done with Promax 3D machine. Cross sectional views with 2 mm slice interval from the left premolar were prepared. The CBCT images revealed the presence of two roots and five root canals (Figure 3). Initially, there was confusion about the presence of two roots or three roots in panoramic radiograph. Periapical radiographs suggested the presence of three roots. The presence of two separate roots in both the sagittal and coronal views of CBCT images aroused suspicions about three roots, but when the axial view was analyzed carefully, it was confirmed that there was a single semilunar root in the coronal and middle thirds, dividing into two portions in the apical third. Irreversible pulpitis and acute apical periodontitis were confirmed, and endodontic treatment was undertaken. The inferior alveolar nerve block was administered with 2% Lidocaine containing 1:80000 epinephrine (Darou Pakhsh, Tehran, Iran). After removal of caries and preparation of the access cavity, the tooth was isolated with a rubber dam. The root canals were explored with an ISO15 K-Flexofile (Dentsply, Maillefer, Switzerland). Five root canals were located, and the morphology was confirmed by radiographic examination (Figure 4).

**Figure 5:**
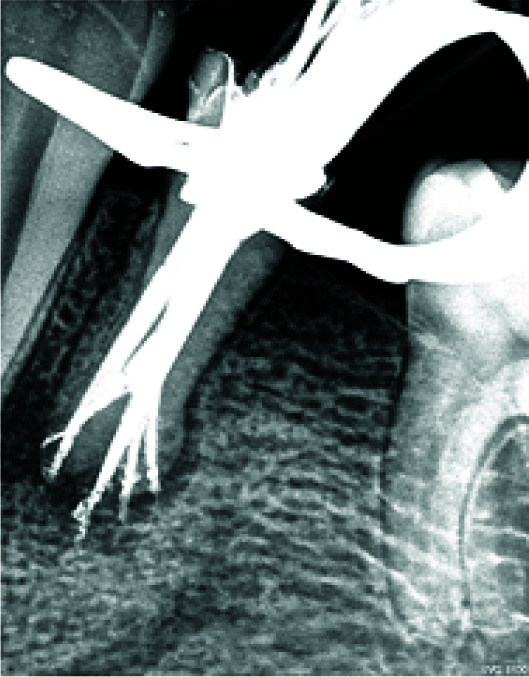
Final radiograph

Working lengths (WL) were established using a Root-ZX apex locator (Morita, Tokyo, Japan), followed by confirmation on a periapical radiograph (Figure 4). The root canals were prepared with RaCe rotary instruments (FKG Dentaire, La Chaux-de-Fonds, Switzerland) up to #25/0.06. Irrigation procedures were carried out with 2.5% NaOCl. Obturation of the root canals was carried out with the lateral condensation technique, using gutta-percha and AH26 sealer (Dentsply, De Trey, Konstanz, Germany). Master apical cone (MAF) was placed in all canals at the same time to prevent blockage of canal entrance during obturation then MAFs were seared off from furcation part using heat carrier then obturation of each canal was done by placing lateral cones in the space which was made by spreader #25 (FKG Dentaire, La Chauxde-Fonds, Switzerland) (Figure 5).

Since there were multiple canals in such small area, it seems that false stop during MAF placement was the reason of overfilling of the canals. The patient was referred to a prosthodontist for restorative and prosthetic procedures.

## Discussion

Mandibular premolars with anatomic variation are the most complicated teeth to perform endodontic treatment [ 4 ]. Possible reasons for these complications are the wide range of anatomic variations that are usually not well realized. The complex nature of the root and root canal system morphology of the lower first premolars has been underestimated [ 3 ]. The majority of these teeth (97.9%) have a single root. Two roots are found in 1.8% of these teeth and three-rooted (0.2%), and four-rooted (0.1%) varieties are very rare [ 3 ]. One root canal is present in 75.8% of the cases, and more root canal systems are present in 24.2% of these teeth [ 3 ]. This case report illustrated the unusual anatomy of the roots and root canal system of a mandibular first premolar in an 18-year-old healthy boy, with three roots, which is quite rare (0.2%), and with five root canals, reported here for the first time. Case reports of mandibular first premolars with three roots and three root canals are rare [ 7 - 8 ], and reports of three or more root canals in second premolars are more frequent [ 9 - 10 ]. The coronal anatomy was within the normal limits, with no indication of the variation in the morphology of the root. Based on CBCT images, the other mandibular first premolar (mandibular right first premolar) had two root canals and two roots. A careful examination of radiographs seems to be necessary when treating mandibular premolars. At least two radiographs with 15° to 20° with either mesial or distal different horizontal angulations are necessary to diagnose multiple root canals in premolar teeth reliably [ 3 ]. Sudden narrowing of the main canal on a parallel radiograph indicates root canal multiplicity [ 11 ]. However, Martinez-Lozano et al. [ 12 ] recommended 40° mesial angulation from the horizontal line as more reliable in finding root canal multiplicity. Moreover, 15° to 30° vertical angular deviation in periapical radiographs is only effective for visualization of root canal system anatomy of mandibular premolars [ 3 ]. Radiographs are two-dimensional representation of three-dimensional objects, with unfavorable superimpositions. Interpretations made using two-dimensional radiographs might provide the clinician with information about the unusual anatomy, without showing the different morphological structures and their interrelations [ 13 ]. Hoen and Pink [ 14 ] found that 42% of teeth needing retreatment had missed roots or root canals. Therefore, it is crucial that all the root canals be identified and debrided during the root canal therapy. CBCT is an extremely useful tool for assessing complex and doubtful anatomies. The value of CBCT in the study of the morphology of complex premolar cases is increasing in clinical endodontics [ 15 - 16 ]. A study concluded that CBCT was superior to other techniques in the identification of multiple root canals in the mandibular first premolars [ 17 ]. CBCT might eventually find routine clinical applications in root canal treatment [ 18 - 19 ]. This case report was written after obtaining informed consent from the patient.

## Conclusion

Dental clinicians should gain adequate knowledge about the anatomy of the root canal system and its diversities and carry out proper radiographic evaluations before and during root canal treatment. The mandibular first premolar teeth might have extremely complex root and canal system morphology, leading to problems during root canal treatment. CBCT is a valuable diagnostic tool in studying the variations of root and root canal system.

## References

[1] England MC Jr, Hartwell GR, Lance JR ( 1991). Detection and treatment of multiple canals in mandibular premolars. J Endod.

[2] Ingle JI ( 1961). A standardized endodontic technique utilizing newly designed instruments and filling materials. Oral Surg Oral Med Oral Pathol.

[3] Cleghorn BM, Christie WH, Dong CCS ( 2007). The root and root canal morphology of the human mandibular first premolar: a literature review. J Endod.

[4] Slowey RR ( 1979). Root canal anatomy. Road map to successful endodontics. Dent Clin North Am.

[5] Rotstein I, Ingle JI (2019). Ingle’s Endodontics.

[6] England MC Jr, Hartwell GR, Lance JR ( 1991). Detection and treatment of multiple canals in mandibular premolars. J Endod.

[7] Chan K, Yew SC, Chao SY (1992). Mandibular premolar with three root canals-two case reports. Int Endod J.

[8] Fischer GM, Evans CE ( 1992). A three-rooted mandibular second premolar. Gen Dent.

[9] Glassman GD ( 1987). Flare-up with associated paresthesia of a mandibular second premolar with three root canals. Oral Surg Oral Med Oral Pathol.

[10] Sachdeva GS, Ballal S, Gopikrishna V, Kandaswamy D ( 2008). Endodontic management of a mandibular second premolar with four roots and four root canals with the aid of spiral computed tomography: a case report. J Endod.

[11] Yoshioka T, Villegas JC, Kobayashi C, Suda H ( 2004). Radiographic evaluation of root canal multiplicity in mandibular first premolars. J Endod.

[12] Martinez-Lozano MA, Forner-Navarro L, Sanchez-Cortes JL ( 1999). Analysis of radiologic factors in determining premolar root canal systems. Oral Surg Oral Med Oral Pathol Oral Radiol Endod.

[13] Holtzman L ( 1998). Root canal treatment of mandibular second premolar with four root canals: a case report. Int Endod J.

[14] Hoen MM, Pink FE ( 2002). Contemporary endodontic retreatments: an analysis based on clinical treatment findings. J Endod.

[15] Cleghorn BM, Christie WH, Dong CCS ( 2008). Anomalous mandibular premolars: a mandibular first premolar with three roots and a mandibular second premolar with a C‐ shaped canal system. Int Endod J.

[16] Nouroloyouni A, Basser R, Salehi ZH, Farhang R, Zadfattah F, Aghajani M ( 2019). Evaluating the Iatrogenic Errors and the Quality of Root Canal Treatment of Mandibular Premolars in Ardabil Population Using the Cone Beam Computed Tomography in 2018. Avicenna J Dent Res.

[17] Matherne RP, Angelopoulos C, Kulild JC, Tira D ( 2008). Use of cone-beam computed tomography to identify root canal systems in vitro. J Endod.

[18] Cotton TP, Geisler TM, Holden DT, Schwartz SA, Schindler WG ( 2007). Endodontic applications of cone-beam volumetric tomography. J Endod.

[19] Nair MK, Nair UP ( 2007). Digital and advanced imaging in endodontics: a review. J Endod.

